# Evaluation by electronic patient-reported outcomes of cancer survivors’ needs and the efficacy of inpatient cancer rehabilitation in different tumor entities

**DOI:** 10.1007/s00520-021-06123-x

**Published:** 2021-03-23

**Authors:** Thomas Licht, Alain Nickels, Gerhard Rumpold, Bernhard Holzner, David Riedl

**Affiliations:** 1 Onkologisches Rehabilitationszentrum St. Veit im Pongau, St. Veiter St. 48, A-5621 St. Veit im Pongau, Austria; 2 Ludwig Boltzmann Institute for Rehabilitation Research, Vienna, Austria; 3 Department of Psychiatry, Psychotherapy and Psychosomatics, Medical University of Innsbruck, Innsbruck, Austria

**Keywords:** Psycho-oncology, Depression, Anxiety, Fatigue, Quality of life, Return to work

## Abstract

**Objective:**

We investigated cancer survivors’ health-related quality of life (HRQOL), specific deficiencies related to underlying disease or treatment, and benefits of rehabilitation in a large variety of cancer entities.

**Patients and methods:**

Electronic patient-reported outcomes were performed as clinical routine procedures. Cancer survivors underwent a 3-week multidisciplinary inpatient rehabilitation. Twenty-one different cancer entities were analyzed separately before (T0) and by the end (T1) of rehabilitation. HRQOL, symptoms, and functions were assessed with EORTC-QLQ-C30 questionnaire, psychological distress with Hospital Anxiety and Depression Scale (HADS).

**Results:**

Four thousand four hundred one of 5912 rehabilitants were evaluable, having completed both questionnaires at T0 and T1. All function mean scores and HRQOL were lower than in Austrian normal population, while levels of anxiety, depression, and all symptom scores were higher. HRQOL was particularly low in lung, liver, and bladder cancer patients. Maximum anxiety levels were observed in patients with breast and thyroid cancer patients, the highest levels of depression in liver and brain cancer patients. Fatigue was severe in patients with lung, liver, esophageal, bladder cancer, and myeloma patients. Mean scores were also high for pain and insomnia. In the group of all rehabilitants, a highly significant improvement of global HRQOL, anxiety, depression, and all function and symptom scores was observed at T1 (*p* < 0.001). We noted significant improvement of HRQOL, anxiety, depression, fatigue, emotional, social, role, and physical functions in each cancer entity with medium to large effect sizes. Other recorded symptoms were reduced in the majority of cancers.

**Conclusion:**

Rehabilitation effectively improves psychological distress and HRQOL as a part of treatment for various cancers.

## Introduction

Advances in cancer treatment have improved life expectancies and cure rates [1, 2]. Many cancer survivors, however, experience severe adverse effects from chemotherapy, radiotherapy, or surgical procedures. Pain, nausea, vomiting, loss of appetite or weight, diarrhea, decreased muscular strength and endurance capacity, lymphedema, cognitive deficits, sleeping disorders, and fatigue reduce HRQOL [3–5]. Thereby, activities and participation can become permanently impaired. Some patients cannot return to work after cancer treatment or become dependent on care [6]. Furthermore, many cancer patients suffer from depression or anxiety, especially fear of disease progression [7–9].

Cancer rehabilitation is aimed at alleviating the symptoms caused by disease or treatment. Physical and social functions should be restored to the best state possible [4]. Physical performance should be enhanced. Equally important are mental stabilization, improvement of nutrition, and pain control. Therapeutic procedures consist of physical, psycho-educative, emotionally supportive, art, and expression. Other interventions include nutrition, lifestyle interventions, or smoking cessation [10]. Hence, cancer rehabilitation generally follows a multidisciplinary approach as an inpatient or outpatient program.

Physicians may misjudge the severity of psychological distress, which can persist after the completion of antitumoral treatment [11]. In addition, many clinicians tend to underestimate somatic symptoms, associated with patients’ distress and poor HRQOL [12]. To address this discordance, patient-reported outcomes (PROs) have been utilized [13]. Using questionnaires or interviews, they offer insight into the patient’s health status, independently from the physicians’ interpretation. Moreover, PROs have been shown to improve the quality of communication between patients and physicians [14]. Among several screening instruments developed to reveal functions, symptoms, and overall HRQOL of cancer patients, QLQ-C30 of the European Organization for Research and Treatment of Cancer (EORTC) has most widely been used in clinical trials for the assessment of the patients’ physical function [15, 16]. A frequently used, validated questionnaire for evaluation of psychological distress is HADS [17–19].

PROs have also been used to evaluate the treatment success of cancer rehabilitation from the cancer survivors’ point of view. Teichmann reported improvements in physical and psychosocial HRQOL, but not of the patients’ functional status [20]. Reduction of anxiety and depression was achieved in breast cancer patients by inpatient rehabilitation trials in Austria and Germany [21, 22]. A 3-month outpatient rehabilitation program improved physical, emotional, and role functions in comparison with a control group while social and cognitive functions remained the same [23]. In an American study, the improvement of functional independence measure scores was observed, with no difference between solid tumors and hematologic malignancies [24]. We have previously reported on the implementation of electronic PROs as a routine measure for the assessment of patients’ needs in a cancer rehabilitation program [25]. The aim of our investigation is to use routine data from patient care for analysis of the impact of cancer rehabilitation on HRQOL, distress, and somatic symptoms of cancer survivors. In an early stage of the current study, we were able to show that psychological distress and HRQOL of cancer survivors improved during a 3-week inpatient rehabilitation measure. With the continuation of this investigation for more than 5 years, we have largely expanded our database. This allows us now to investigate the rehabilitants’ specific needs with respect to their underlying disease, and the outcomes of cancer rehabilitation in different cancer entities.

## Patients and methods

Data was collected as a part of clinical routine procedures at the Oncological Rehabilitation Center St. Veit im Pongau, Austria. Adult cancer survivors underwent inpatient rehabilitation measures, with costs being covered by the Austrian pension funds. Rehabilitation lasted 21 days with 2–3 h of therapeutic units per working day. The patients’ stays could be extended for another 7 days in case of severe impairment.

Baseline assessment (T0) was performed prior to admission to the rehabilitation center. To this end, patients were provided with an access code that enabled them to complete questionnaires at home and submit them online. Submitted information was used for planning therapeutic procedures and allocating resources. The assessment of functions, symptoms, and of psychological distress was conducted using QLQ-C30 and HADS, respectively. QLQ-C30 consists of 30 questions, building a scale for global HRQOL, 5 functioning (physical, social, role, emotional, cognitive), and 9 symptom scales (fatigue, nausea/vomiting, pain, dyspnea, sleep disturbances, appetite loss, constipation, diarrhea, financial impact). Scoring was undertaken according to the EORTC scoring manual with raw scores being transformed to a scale from 0 to 100 [15]. Here, 100 reflects the worst symptom score and the best functioning score. HADS consists of 14 items that are used for calculation of a total score ranging from 0 to 42 (0–21 for the anxiety and depression subscales, respectively). Clinical cases of anxiety or depression are identified by scores of 11 or greater, while cases with scores from 7 to <11 are considered doubtful [18].

At the time of the admission, patients were asked by physicians or psychologists whether they were willing to participate in an observational study. Upon written informed consent, they were included in the study for evaluation of treatment success. The study had been submitted to the Ethics Commission of the state of Salzburg (no. 415-EP/73/451-2014) and was conducted according to the principles of the Declaration of Helsinki. Participants of the study completed the abovementioned questionnaires again by the end of rehabilitation (T1). Data was gathered and analyzed with the use of the Computer-Based Health Evaluation Software (CHES), which has been described [26]. The current investigation took place from August 2014 until end of September 2018. In the case of repeated rehabilitations, only the first stay of one patient was included. Further exclusion criteria included early termination of the rehabilitation within 3 days; extended interval >56 days between T0 and the start of rehabilitation; and incomplete data (missing T0 or T1).

### Treatment measures during the rehabilitation

Patients received multidisciplinary therapies including guidance and treatment by physicians, nursing, physiotherapy, aerobic and resistance training, psychological counseling, biofeedback or relaxation exercises, nutritional advice, social counseling, and educational presentations including motivation to lifestyle modifications. Most patients were also treated with occupational therapy, remedial massages, thermotherapy, hydrogymnastics, electrotherapy, or offered counseling for smoking cessation. The frequency of therapeutic units is displayed in Table 1. The minimum time of all treatment measures was at least 1800 min within 21 days. The guidelines of the Austrian pension funds, which require certain frequencies for the respective therapies, served a basis for the treatment planning.

**Table 1:** Treatment modalities. Overview over therapeutic measures per patient during the rehabilitation. *n*: number of patients who received specific treatment at least once (percentage of patients of the whole collective); IQR: interquartile range

Treatment modality			Treatment frequency per patient	
	*n*	(%)	Median	IQR
Guidance and treatment by physician	4401	(100.0%)	6	6–7
Nursing procedures	4392	(99.8%)	2	2–2
Psychooncology (individual counseling) including biofeedback	4195	(95.3%)	5	4–7
Psychooncological counseling (group)	4401	(100.0%)	1	1–1
Psychological counseling: sexual therapy	798	(18.1%)	3	1–5
Psychoeducative lectures	4393	(99.8%)	3	3–4
Relaxation therapies	4400	(99.9%)	4	3–5
Educational presentations—motivation and lifestyle modification	4182	(95.0%)	2	1–3
Educational lectures	3882	(88.2%)	1	1–2
Cognitive and perception training	1048	(23.8%)	2	2–4
Creative therapies	597	(13.6%)	2	2–4
Social counseling	3719	(84.5%)	2	1–2
Speech therapy	331	(7.5%)	4	2–6
Nutritional advice	4399	(99.9%)	4	3–4
Occupational therapy (individual treatment)	3579	(81.3%)	2	2–3
Functional occupational therapies (groups)	4150	(94.3%)	7	4–7
Physiotherapy (individual treatment)	4399	(99.9%)	6	5–8
Physiotherapy (groups)	4373	(99.4%)	7	5–10
Medical training therapy—aerobic training	4355	(99.0%)	7	5–9
Medical training therapy— resistance training	3911	(88.9%)	5	4–6
Remedial massages	4207	(95.6%)	4	3–4
Manual lymphatic drainage	1192	(27.1%)	4	2–5
Hydrogymnastics	2014	(45.8%)	3	2–4
Electrotherapy	2165	(49.2%)	4	3–6
Therapeutic ultrasound	256	(5.8%)	3	2–5
Thermotherapy	3416	(77.6%)	5	3–7
Inhalation therapies	682	(15.5%)	4	5–7

### Statistical analyses

Changes of psychological distress, HRQOL, and subjective working ability as a result of the rehabilitation were analyzed using repeated measures analysis of variance (ANOVA). Analyses were conducted for the total collective and for 21 cancer entities separately. Partial eta squared (*η*^*2*^) was calculated to estimate the effect size of the mean differences in regard to psychological distress, symptoms, and HRQOL. Values of *η*2 = 0.01, *η*2 = 0.06, and *η*2 = 0.14 were considered as small, medium, and large, respectively [27].

Additionally, QLQ-C30 differences of 5–10 points, 10–20 points, and >20 point indicated small, moderate, or large changes, respectively [28]. A minimal important difference for the HADS anxiety and depression score of 1.3 and 1.4 points, respectively, has been described [29]. Mean values at T0 and T1 were compared to reference values for the QLQ-C30 and the HADS [30, 31]. *P* values <0.05 were considered statistically significant. All calculations were conducted with SPSS (v21).

## Results

Of the initial sample of 5912 patients, 4401 patients (74.4%) were evaluable for the current investigation. Fourteen patients (0.2%) were excluded because of the early termination of the rehabilitation within the first 3 days, and 424 patients (7.2%) because of incomplete data (missing T0 or T1). Furthermore, in 451 cases (7.6%), the interval between T0 and rehabilitation was >56 days; thus, the T0 status was considered likely to have changed in between. Six hundred twenty-two cases (10.5%) were admissions of identical patients for repeated rehabilitation procedures, which might have biased the study.

As displayed in Table 2, most patients showed medium levels of performance (Karnofsky Performance Status 50–80%: 62.8%, ECOG Status 1: 55.6%). The medium age was 61.3 years, and the majority of patients were female. Twenty-one distinct cancer entities were analyzed separately, comprising >94% of rehabilitants. Thereby, the different sub-entities of head and neck cancers; malignant lymphomas; leukemias; and uterine cancers were grouped together. More than 50% of the patients had been diagnosed with cancers originating from breast, colon, rectum, or prostate, i.e., the most frequent tumors in the general population. Hematologic malignancies were slightly above 10%.

**Table 2:** Patient characteristics. Patients were admitted to the rehabilitation measures between August, 2014, and end of September, 2018. Shown are mean values and standard deviation (SD) of sociodemographic and clinical data. Assignment of cancer entities was performed according to the respective ICD-10 codes (German Modification) of the primary diagnoses

Sample size	4401	
Mean age (SD)	61.3 yr	(SD 12.0)
Range	31–90 yr	
Sex		
Male	1653	37.6%
Female	2746	62.4%
Missing information	2	0.05%
Mean body mass index/BMI (SD)	25.7 kg/m^2^	(5.1)
Range	14.4–53.4 kg/m^2^	
Smokers	751	17.1 %
Karnofsky Performance Score		
High level of functioning (80–100%)	1,556	35.4%
Medium level of functioning (50–80%)	2,763	62.8%
Low level of functioning (0–50%)	26	0.6%
Missing information	56	1.3%
ECOG score		
Grade 0	368	8.4%
Grade 1	2,447	55.6%
Grade 2	1,457	33.1%
Grade 3	58	1.3%
Missing information	71	1.6%
Cancer entities		
Head and neck cancers (C00-14; C30-C32)	232	5.3%
Esophageal cancer (C15)	64	1.5%
Gastric cancer (C16)	120	2.7%
Colon cancer (C18-19)	261	5.9%
Rectal cancer (C20-21)	151	3.4%
Liver cancer (C22)	35	0.8%
Pancreatic cancer (C25)	99	2.2%
Lung cancers (C33-C34)	219	5.0%
Skin cancers (C43-44)	41	0.9%
Breast cancer (C50)	1,534	34.9%
Uterine cancers (C51-55)	140	3.2%
Ovarian cancer (C56)	152	3.5%
Prostate cancer (C61)	323	7.3%
Testicular cancer (C62)	43	1.0%
Renal cancer (C64)	86	2.0%
Bladder cancer (C67)	99	2.2%
Brain cancers (C70-72)	59	1.3%
Thyroid cancer (C73)	45	1.0%
Malignant lymphomas (C81-C86; C88)	299	6.8%
Multiple myeloma (C90)	72	1.6%
Leukemias (C91-C95)	79	1.8%
Other cancer types	248	5.6%

### Assessment of cancer survivors’ needs

The baseline assessment (T0) was used to determine the needs and particular symptoms of survivors from different cancers. We wished to understand the specific deficiencies with respect to the underlying cancer entities. Analysis of global HRQOL with the QLQ-C30 instrument revealed substantially decreased mean function scores in the whole patient group as well as in each cancer entity (Table 3). Reduced HRQOL is an indication for the implementation of a rehabilitation measure. Mean HRQOL of all rehabilitants was 57.6 compared to 75.65, the reported mean HRQOL in the common Austrian population [30]. We found HRQOL was particularly low in patients with cancers of lung, liver, or urinary bladder.

**Table 3:**
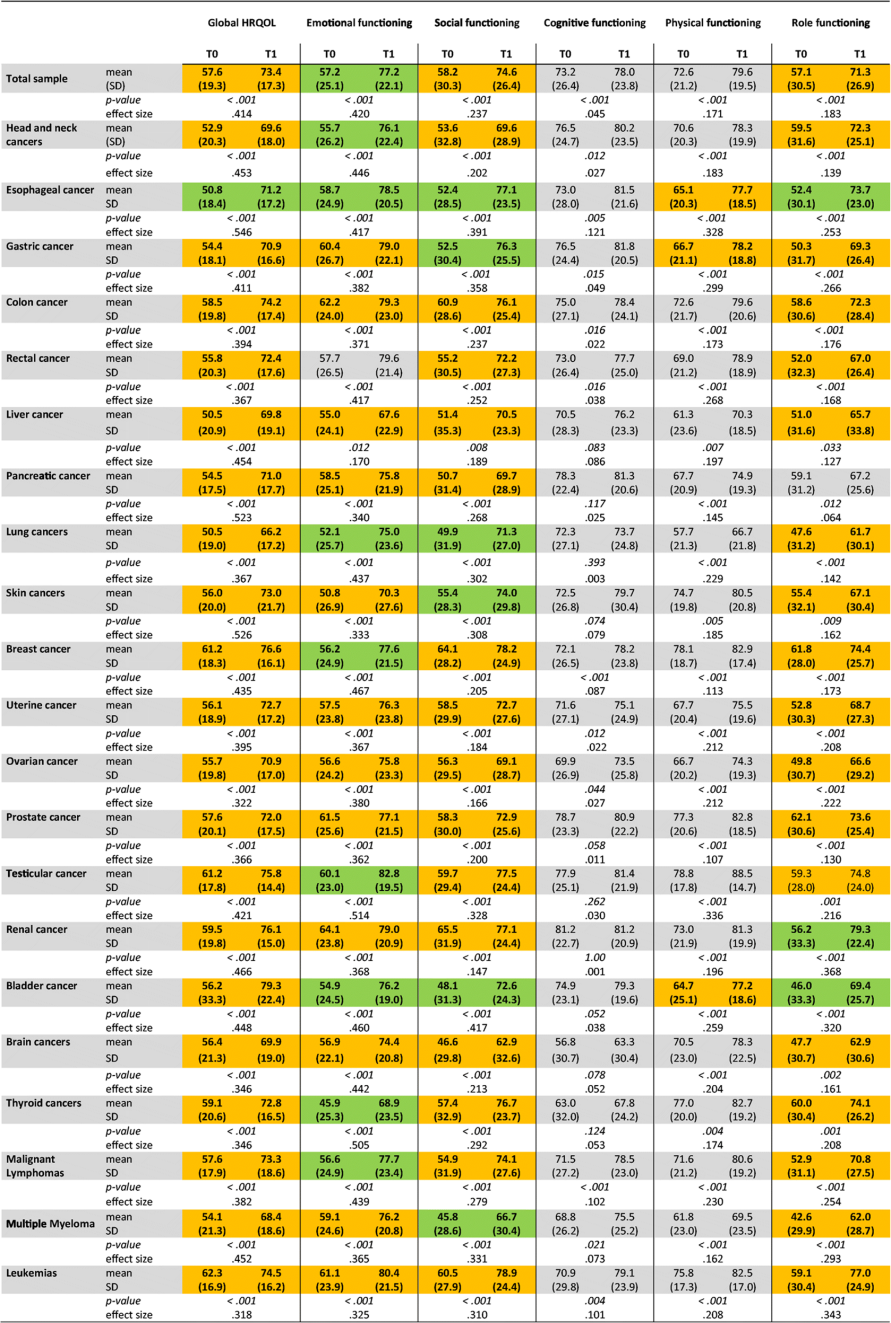
HRQOL and functioning scores in the whole group of 4,401 cancer survivors, and in different cancer entities. Shown are mean scores prior to (T0), and by the end (T1) of the rehabilitation measures as assessed by EORTC QLQ-C30, standard deviation (SD), significance (*p*), and effect size (*η*^2^). Effect size is considered small for *η*^2^ > 0.01, medium for *η*^2^ > 0.06, and large for *η*^2^ > 0.14. Mean differences between T0 and T1 of >10 points (moderate change) are highlighted in yellow, and of >20 points (large changes) in green [28]

Psychological distress was determined with HADS. Each group of cancer survivors suffered from anxiety and depression (Table 4) compared with the average German population, which has been reported [19]. Patients with lung, thyroid, or brain cancers were particularly distressed, showing high levels of both anxiety and depression. Elevated anxiety levels were also noticed in patients with cancers of breast, uterus, and ovary. Patients with liver, bladder, head and neck, or prostate cancer were severely depressed.

**Table 4:**
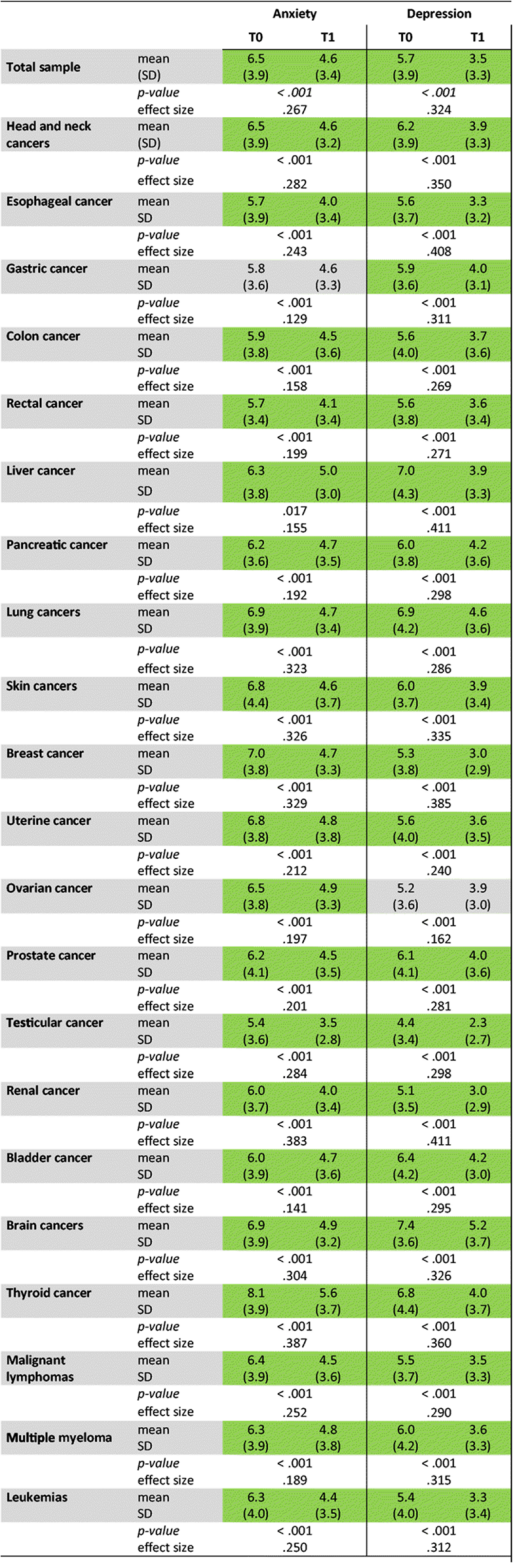
Psychological distress in the whole group of cancer survivors, and in different cancer entities. Shown are mean scores of anxiety and depression at T0 and T1 as assessed by HADS. Standard deviation (SD), significance (*p*), and effect size (*η*^2^) are displayed. Effect size is considered small for *η*^2^ > 0.01, medium for *η*^2^ > 0.06, and large for *η*^2^ > 0.14. Mean differences between T0 and T1 greater than the cut-off levels for clinical relevance are highlighted in green (i.e., for anxiety: 1.3 points; for depression: 1.4 points [29])

The impairment of emotional, role, and social functions was generally more pronounced than of physical and cognitive functions (Table 5). For the social function, the difference between the cohort of cancer survivors and the normal Austrian population was most striking (QLQ-C30 mean scores 58.2 vs. 92.23) [30]. The lowest mean scores for social and role functions were observed in patients with multiple myeloma, brain, bladder, and lung cancer. While the emotional function was notably low in patients with cancers originating from lung, skin, or thyroid gland, it was moderately good in patients with renal, colon, and prostate cancer and leukemia. Social function was least impaired in breast and renal cancer survivors, and role function in breast, prostate, and thyroid cancer. Survivors of lung or liver cancers, and myeloma revealed particularly poor physical function, while patients with brain or thyroid cancers felt a major negative impact of cognitive limitation. In contrast, the physical function reported by breast, prostate, testicular, and thyroid cancer patients was fairly good.

**Table 5:**
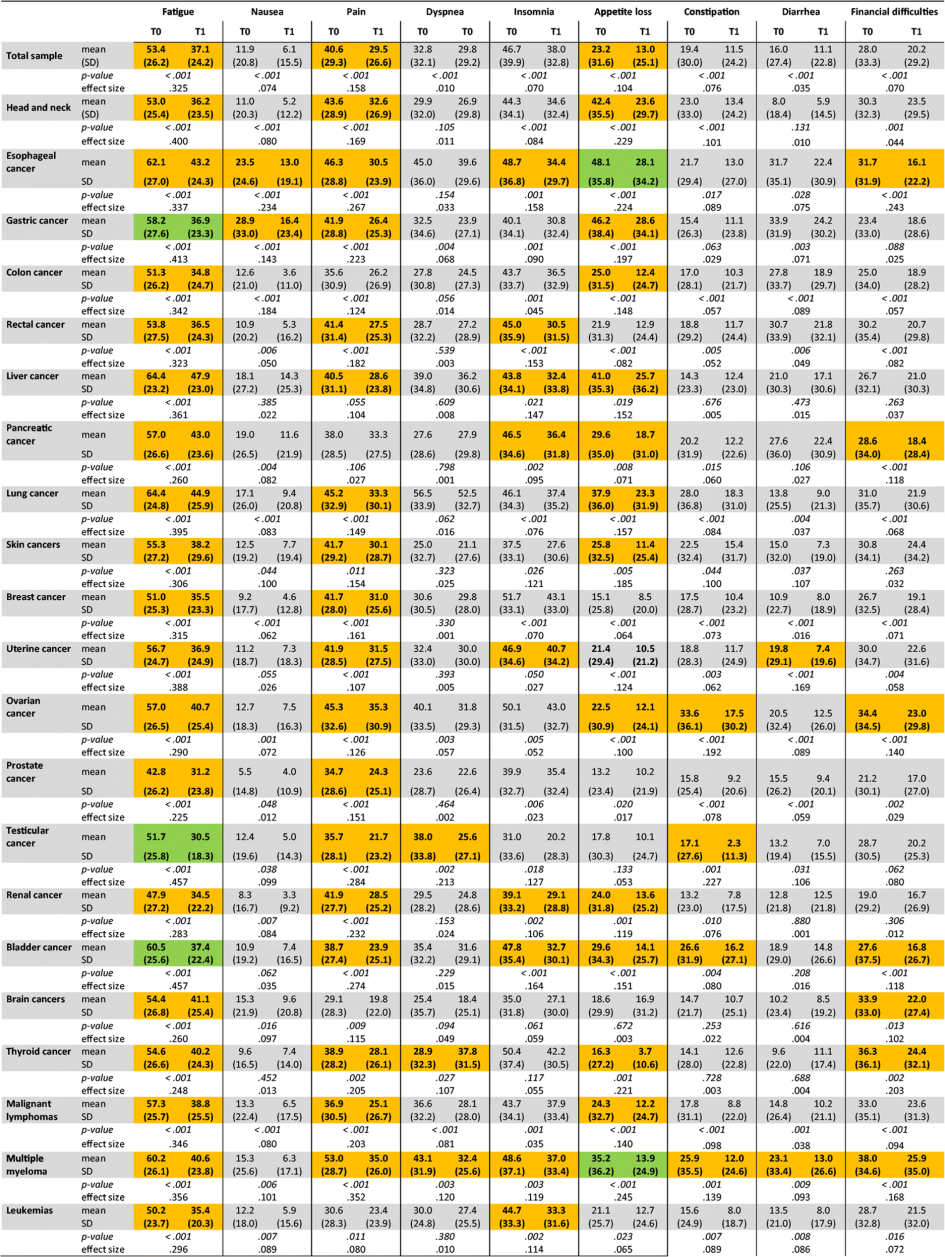
Symptom scores in the whole group cancer survivors, and in different cancer entities. Shown are mean scores at T0 and T1 as assessed by EORTC QLQ-C30, standard deviation (SD), significance (*p*), and effect size (*η*^2^). Effect size is considered small for *η*^2^ > 0.01, medium for *η*^2^ > 0.06, and large for *η*^2^ > 0.14. Mean differences between T0 and T1 of >10 points (moderate change) are highlighted in yellow, and of >20 points (large changes) in green [28]

Fatigue, sleeping disorders, and pain were prominent among the rehabilitants. For fatigue, the mean score was as high as 53.4 in the whole group of patients with cancers of lung, liver, esophagus, and bladder, and myeloma patients being most affected (Austrian population: 24.12). Conversely, prostate and renal cancer patients had lower scores for fatigue. Insomnia was fairly common with highest levels in breast, ovarian, and thyroid cancer survivors, but not in testicular, skin, or brain cancer survivors. Esophageal, ovarian, and lung cancer patients were severely pain-stricken, while esophageal and lung cancer patients suffered most from dyspnea. In contrast, brain cancer and leukemia patients were least affected by pain, and dyspnea was uncommon in patients with skin and prostate cancer. Gastrointestinal symptoms were generally described as less severe. Loss of appetite was mainly observed in patients with gastric, esophageal, hepatic, and head and neck cancers. Survivors of ovarian and lung cancer reported constipation. Survivors of cancers originating in stomach, esophagus, or rectum, and to lesser degree of colon, pancreas were impeded by diarrhea. Financial impact is also recorded by the QLQ-C30 instrument. Myeloma, ovarian, and thyroid cancer patients were most worried because of financial difficulties.

### Improvement of functions and global HRQOL by rehabilitation

In the whole group of rehabilitants and each investigated cancer type, highly significant improvement of global HRQOL was observed by the end of the rehabilitation measure (Table 3). More importantly, effect sizes were large for all entities as determined by *η*^*2*^. Furthermore, emotional, social, role, and physical functions were significantly improved in all cancer entities (Table 3). The effect sizes were large for role function in most cancers, except for patients with head and neck, prostate, and liver cancers, where effects were medium size. The increase of mean scores exceeded 20 points of the QLQ-C30 scores, indicating major improvement: [28] for emotional function in patients with cancers of lung, head and neck, thyroid gland, breast, esophagus, bladder, testes, rectum, and in malignant lymphomas; for social function in myeloma, and gastric, esophageal, lung, skin, and bladder cancer patients; for role function in patients with esophageal, renal, and bladder cancer. The effect sizes for improvement of the physical function were large in patients of all cancer types except for breast and prostate cancer where they were medium size. Cognitive function was improved in 12 of 21 cancer entities. The effect was, however, less pronounced.

### Reduction of psychological distress

We found depression significantly reduced with large effect sizes in the whole group (Table 4). The effect on anxiety was also large in all cancer types, except for patients with gastric cancer where it was of medium size. Those patient groups that were particularly distressed before showed a major benefit, e.g., patients with breast, thyroid, and lung cancer.

The reduction of HADS scores for anxiety and depression by 1.9 and 2.2, respectively, is considered a clinically meaningful improvement [29]. Such improvement was found for the whole collective and most cancer entities. Remarkably, the anxiety mean score for all patients was lowered to the normal range of the German population, and the score of depression was diminished even below this level [19, 31]. Depression improved particularly well for rehabilitants suffering from liver, brain, lung, and thyroid cancer.

### Alleviation of symptoms

All symptom scores were decreased in the whole group of rehabilitants (Table 5). Fatigue was ameliorated with generally large effect sizes in patients of all cancer types, most remarkably in survivors of gastric, lung, uterine cancer, myeloma, and malignant lymphomas. Similarly, pain and appetite loss were significantly reduced in all but two cancer entities. Dyspnea was improved in patients with gastric, ovarian, testicular, thyroid cancers, lymphomas, and myelomas. All other symptoms including gastrointestinal complaints were reduced in the majority of tumor entities. Significant worsening of symptoms was not noted. In addition, financial worries were reduced in the majority of cancer patients.

### Self-assessed ability to work

**Fig. 1:**
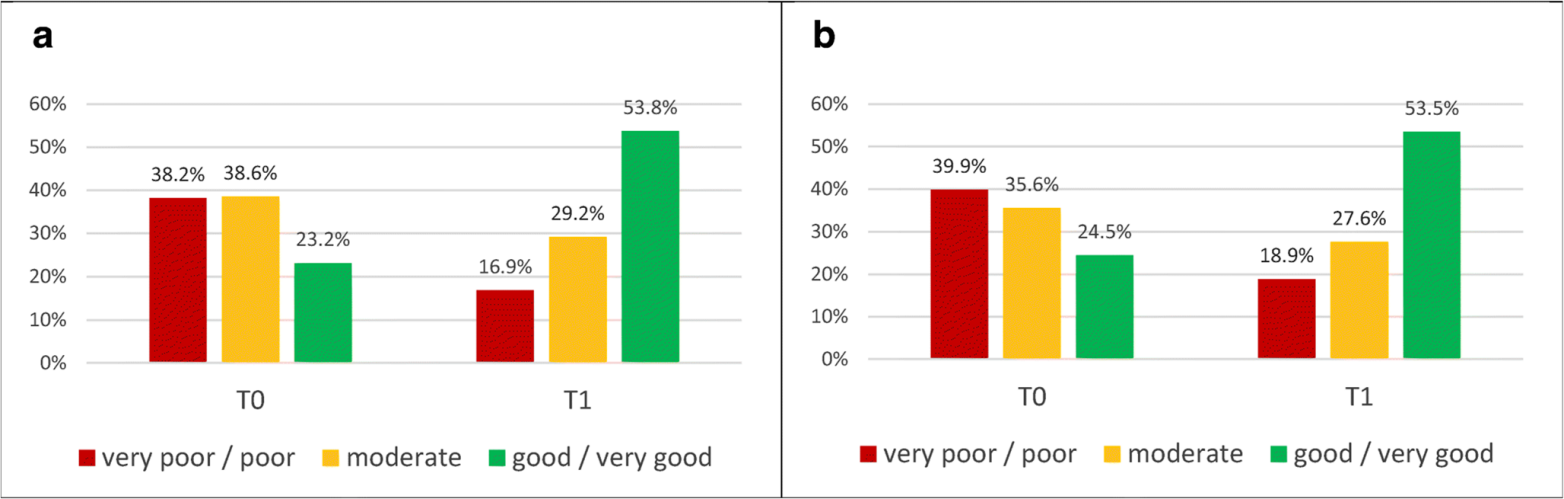
Self-assessed ability to work. Patients were asked to assess their ability to work by T0 and T1. Shown are percentages of patients who believed they were capable of working full-time, part-time, or unfit to work. **a** All rehabilitants who have stated their working capability (*n* = 1973). **b** Rehabilitants < 65 years of age, belonging to the work force (*n* = 1272)

We also asked the patients whether they felt capable of working. The number of rehabilitants who thought they were able to return to work was <25% prior to the rehabilitation measure (Fig. 1a). This number was more than doubled by T1. Almost identical results were obtained when only analyzing the subgroup of rehabilitants <65 years of age (Fig. 1b).

## Discussion

We have analyzed the impairments of cancer survivors and the benefits of an inpatient rehabilitation measure. The high number of participants in this observational study enabled us to retrospectively analyze a broad variety of cancer entities with respect to psychosocial and somatic burden. To our best knowledge, this is the largest investigation of the effects of rehabilitation in different cancer entities with the use of PROs. While reduced global HRQOL and psychological distress are present in all patient groups, we noticed apparent differences due to the respective underlying neoplasms. The frequently poor prognosis [1], and low function scores of patients with brain or lung cancer are reflected by high levels of distress. However, high levels of psychological distress were not restricted to patients suffering from cancers with a generally dismal prognosis. Of note, anxiety was lower in patients with pancreatic, bladder, ovarian, or liver cancer than in patients with thyroid or breast cancer despite considerably poorer prognosis [1, 2]. The very high level of distress in thyroid cancer survivors is in agreement with previous reports [32]. Prognosis, however, is mostly favorable regarding this cancer, and cure rates are high [2]. The high burden of psychological distress of breast cancer survivors has been described. Mehnert reported that 23.6% of breast cancer patients were classified as having moderate to high fear of tumor progression, and high negative correlation of fear and HRQOL was reported [33]. We found that breast cancer survivors suffer severely from psychological distress despite comparably good physical, social, and role functions. This observation contrasts studies that describe the limitations of physical, role, emotional, cognitive, and social functioning limitations as predictors of distress in female breast cancer survivors [34].

There was also some discrepancy between somatic symptoms and psychological distress. Despite experiencing pain, gastrointestinal symptoms, or dyspnea, patients with ovarian, esophageal, or gastric cancers reported intermediate levels of psychological distress. Rehabilitation of these patients should focus on physical deficiencies and nutrition while psychosocial counseling should have priority in breast cancer. The drivers of psychological burden, and the strategies for coping and disease processing deserve further attention across certain tumor entities. Our findings may contribute to the development of rehabilitation programs tailored to distinct needs in certain cancers.

In accordance with other trials [10], our rehabilitation program was multidisciplinary, integrating physical, psycho-oncological, and educative components including life-style modification and nutrition. These components contribute to changes of different functions and symptoms. In accord with meta-analyses, we find very high symptom scores for fatigue [35], sleeping disorders, and pain in our group of cancer survivors. Fatigue is considered the most commonly reported and distressing symptom in cancer patients. With evidence-based treatment modalities for fatigue including physical activity, aerobic and resistance training, massage, and relaxation [35, 36], major improvements of fatigue could be observed in our study. Patients of each cancer entity benefited from rehabilitation. Furthermore, the importance of exercise for improvement of global HRQOL, physical function, and fatigue is established [37]. Pain, fatigue, and sleep disturbances can be reduced by cognitive-behavioral strategies [38], which were one component of psycho-oncological counseling and educational interventions. The evidence for psychological interventions for fatigue after cancer is considered tentative [39]. Psychosocial interventions have been shown to mainly improve emotional and social functions, and global HRQOL [40]. Furthermore, strength and physical function, which are diminished by cancer therapy, are improved by oncologic rehabilitation [41].

Our study has several limitations. This is not a representative study of all cancer survivors, but restricted to those persons who were capable of and felt the need for rehabilitative measures. The propensity to apply for a rehabilitative measure may be different among patients due to age, gender, or advice by support groups. The distribution of cancer entities may thus differ from the prevalence in the general population. Moreover, severely disabled patients in a palliative setting were incapable of participating in the rehabilitative measures. Some patients had to terminate early within few days, and others had to be transferred to hospitals because of acute health complications. These patients could not be included into this study. Furthermore, a comprehensive analysis of the medical history and socio-demographic status was not feasible in our study. This data is not documented in a standardized fashion but rather paraphrased by the physicians.

Several reports have previously described improvement of various components of HRQOL by inpatient or outpatient cancer rehabilitation [21–25, 42]. Many investigations focus on patients from certain cancer entities, such as breast [21, 22, 42], bladder [43], or prostate cancer [44]. In contrast, our aim was to compare the effects of oncological rehabilitation in a spectrum of patients from major cancer entities.

An interesting finding of our study is the improved self-assessed ability to work by the end of the rehabilitation measure. It is known that cancer survivors are less likely to be employed and take more sick leave than workers without a history of cancer [45]. Hence, early retirement and non-employment are common among cancer survivors [6]. Improved self-assessed capability of returning to work may help avoid unemployment. It might also be related to self-esteem and participation.

The durability of the benefits accomplished by rehabilitation has been shown by others. In a large study with 3233 patients, Klocker demonstrated that inpatient rehabilitation significantly improved HRQOL, anxiety, and depression, which persisted after 6 and 12 months [22]. We have started a survey that includes follow-up evaluations 3, 6, and 12 months after the rehabilitation measure. Similarly, HRQOL and symptoms were stably improved after a 3-month outpatient exercise and education program during 2 years of follow-up [42]. Further studies should determine whether certain cancer patients require repeated rehabilitation measures to conserve their HRQOL and avoid unemployment.

## Conclusion

Psychological distress with anxiety and depression, and fatigue are common among cancer survivors. A 3-week, multidisciplinary cancer rehabilitation measure can significantly alleviate these and most other symptoms in all 21 investigated cancer entities. All functions are markedly improved in the majority of cancers. In summary, cancer rehabilitation is highly effective in improving the quality of life of cancer survivors. The identification of the specific needs according to the underlying malignant diseases may help design specific rehabilitation programs.

## Data Availability

Data are part of the patients’ medical records at Onkologisches Rehabilitationszentrum St. Veit im Pongau. They have been anonymized for statistical analysis.

## Code availability

Computer-based health evaluation software (CHES) has been used for analysis of the data. This software application is described under: https://ches.pro/.

## References

[1] DeAngelis R, Sant M, Coleman MP (2014). Cancer survival in Europe 1999–2007 by country and age: results of EUROCARE-5 - a population-based study. Lancet Oncol.

[2] Miller KD, Nogueira L, Mariotto AB, Rowland JH, Yabroff KR, Alfano CM, Jemal A, Kramer JL, Siegel RL (2019). Cancer treatment and survivorship statistics, 2019. CA Cancer J Clin.

[3] Dong ST, Butow PN, Costa DS (2014). Symptom clusters in patients with advanced cancer: a systematic review of observational studies. J Pain Symptom Manag.

[4] Maehr B, Keilani M, Wiltschke C, Hassler M, Licht T, Marosi C, Huetterer E, Cenik F, Crevenna R (2016). Cancer rehabilitation in Austria - aspects of physical medicine and rehabilitation. Wien Med Wochenschr.

[5] Strömgren AS, Sjogren P, Goldschmidt D, Petersen MA, Pedersen L, Groenvold M (2006). Symptom priority and course of symptomatology in specialized palliative care. J Pain Symptom Manag.

[6] Lindbohm SL, Kuosma E, Taskila T (2014). Early retirement and non-employment after breast cancer. Psychooncology.

[7] Reed SC, Bell JF, Whitney R, Lash R, Kim KK, Bold RJ, Joseph JG (2018). Psychosocial outcomes in active treatment through survivorship. Psychooncology.

[8] Carlson LE, Angen M, Cullum J, Goodey E, Koopmans J, Lamont L, MacRae JH, Martin M, Pelletier G, Robinson J, Simpson JSA, Speca M, Tillotson L, Bultz BD (2004). High levels of untreated distress and fatigue in cancer patients. Br J Cancer.

[9] Hartung TJ, Brähler E, Faller H, Härter M, Hinz A, Johansen C, Keller M, Koch U, Schulz H, Weis J, Mehnert A (2017). The risk of being depressed is significantly higher in cancer patients than in the general population: prevalence and severity of depressive symptoms across major cancer types. Eur J Cancer.

[10] Gudbergsson SB, Dahl AA, Loge JH, Thorsen L, Oldervoll LM, Grov EK (2015). What is covered by “cancer rehabilitation” in PubMed? A review of randomized controlled trials 1990–2011. J Rehabil Med.

[11] Passik S, Dugan W, McDonald MV, Rosenfeld B, Theobald DE, Edgerton S (1998). Oncologists’ recognition of depression in their patients with cancer. J Clin Oncol.

[12] Chandwani KD, Zhao F, Morrow GR, Deshields TL, Minasian LM, Manola J, Fisch MJ (2017). Lack of patient-clinician concordance in cancer patients: its relation with patient variables. J Pain Symptom Manag.

[13] Wintner LM, Giesinger JM, Kemmler G, Sztankay M, Oberguggenberger A, Gamper EM, Sperner-Unterweger B, Holzner B (2012). Verwendung und Nutzen von Patient-Reported Outcomes in der onkologischen Behandlung: eine Übersicht. Wien Klin Wochenschr.

[14] Detmar SB, Muller MJ, Schornagel JH, Wever LD, Aaronson NK (2002). Health-related quality-of-life assessments and patient-physician communication: a randomized controlled trial. JAMA.

[15] Aaronson NK, Ahmedzai S, Bergman B, Bullinger M, Cull A, Duez NJ, Filiberti A, Flechtner H, Fleishman SB, Haes JCJM, Kaasa S, Klee M, Osoba D, Razavi D, Rofe PB, Schraub S, Sneeuw K, Sullivan M, Takeda F (1993). The European Organization for Research and Treatment of Cancer QLQ-C30: a quality-of-life instrument for use in international clinical trials in oncology. J Natl Cancer Inst.

[16] Atkinson TM, Stover AM, Storfer DF, Saracino RM, D'Agostino TA, Pergolizzi D, Matsoukas K, Li Y, Basch E (2017). Patient-reported physical function measures in cancer clinical trials. Epidemiol Rev.

[17] Zigmont AS, Snaith RP (1983). The hospital anxiety and depression scale. Acta Psychiatr Scand.

[18] Herrmann C (1997). International experiences with the Hospital Anxiety and Depression Scale - a review of validation data and clinical results. J Psychosom Res.

[19] Hinz A, Brähler E (2011). Normative values for the Hospital Anxiety and Depression Scale in the general German population. J Psychosom Res.

[20] Teichmann JV (2002). Onkologische Rehabilitation: Evaluation der Effektivität stationärer onkologischer Rehabilitationsmaßnahmen. Rehabilitation.

[21] Hartmann U, Kluge A, Ring C, Reuss-Borst M (2006). Improvement of anxiety and depression in women with breast cancer during inpatient oncological rehabilitation—results of a prospective study. Rehabilitation.

[22] Klocker J, Klocker-Kaiser U, Pipam W, Geissler D (2018). Long-term improvement of the bio-psycho-social state of cancer patients after 3 weeks of inpatient oncological rehabilitation: A long-term study at the Humanomed Zentrum Althofen. Wien Med Wochenschr.

[23] Leclerc AF, Foidart-Delasselle M, Tomasella M (2017). Multidisciplinary rehabilitation program after breast cancer: benefits on physical function, anthropometry and quality of life. Eur J Phys Rehabil Med.

[24] Guo Y, Shin KY, Hainley S, Bruera E, Palmer JL (2011). Inpatient rehabilitation improved functional status in asthenic patients with solid and hematologic malignancies. Am J Phys Med Rehabil.

[25] Riedl D, Giesinger JM, Wintner LM, Loth FL, Rumpold G, Greil R, Nickels A, Licht T, Holzner B (2017). Improvement of quality of life and psychological distress after inpatient cancer rehabilitation: results of a longitudinal observational study. Wien Klin Wochenschr.

[26] Holzner B, Giesinger JM, Pinggera J (2012). The Computer-based Health Evaluation Software (CHES): a software for electronic patient-reported outcome monitoring. BMC Med Inform Decis Mak.

[27] Ellis PD (2010). The essential guide to effect sizes: statistical power, meta-analysis, and the interpretation of research results.

[28] Osoba D, Rodrigues G, Myles J, Zee B, Pater J (1998). Interpreting the significance of changes in health-related quality-of-life scores. J Clin Oncol.

[29] Puhan MA, Frey M, Büchi S, Schünemann HJ (2008). The minimal important difference of the hospital anxiety and depression scale in patients with chronic obstructive pulmonary disease. Health Qual Life Outcomes.

[30] Lehmann J, Giesinger JM, Nolte S (2020). Normative data for the EORTC QLQ-C30 from the Austrian general population. Health Qual Life Outcomes.

[31] Hinz A, Brähler E (2011). Normative values for the hospital anxiety and depression scale (HADS) in the general German population. J Psychosom Res.

[32] Roerink SH, de Ridder M, Prins J (2013). High level of distress in long-term survivors of thyroid carcinoma: results of rapid screening using the distress thermometer. Acta Oncol.

[33] Mehnert A, Berg P, Henrich G, Herschbach P (2009). Fear of cancer progression and cancer-related intrusive cognitions in breast cancer survivors. Psychoncology.

[34] Syrowatka A, Motulsky A, Kurteva S, Hanley JA, Dixon WG, Meguerditchian AN, Tamblyn R (2017). Predictors of distress in female breast cancer survivors: a systematic review. Breast Cancer Res Treat.

[35] Tian L, Lu HJ, Lin L, Hu Y (2016). Effects of aerobic exercise on cancer-related fatigue: a meta-analysis of randomized controlled trials. Support Care Cancer.

[36] Hilfiker R, Meichtry A, Eicher M, Nilsson Balfe L, Knols RH, Verra ML, Taeymans J (2018). Exercise and other non-pharmaceutical interventions for cancer-related fatigue in patients during or after cancer treatment: a systematic review incorporating an indirect-comparisons meta-analysis. Br J Sports Med.

[37] McNeely ML, Campbell KL, Rowe BH, Klassen TP, Mackey JR, Courneya KS (2006). Effects of exercise on breast cancer patients and survivors: a systematic review and meta-analysis. CMAJ.

[38] Kwekkeboom K, Zhang Y, Campbell T, Coe CL, Costanzo E, Serlin RC, Ward S (2018). Randomized controlled trial of a brief cognitive-behavioral strategies intervention for the pain, fatigue, and sleep disturbance symptom cluster in advanced cancer. Psychooncology..

[39] Corbett TK, Groarke A, Devane D, Carr E, Walsh JC, McGuire BE (2019). The effectiveness of psychological interventions for fatigue in cancer survivors: systematic review of randomised controlled trials. Syst Rev.

[40] Kalter J, Verdonck-de Leeuw IM, Sweegers MG, Aaronson NK, Jacobsen PB, Newton RU, Courneya KS, Aitken JF, Armes J, Arving C, Boersma LJ, Braamse AMJ, Brandberg Y, Chambers SK, Dekker J, Ell K, Ferguson RJ, Gielissen MFM, Glimelius B, Goedendorp MM, Graves KD, Heiney SP, Horne R, Hunter MS, Johansson B, Kimman ML, Knoop H, Meneses K, Northouse LL, Oldenburg HS, Prins JB, Savard J, van Beurden M, van den Berg SW, Brug J, Buffart LM (2018). Effects and moderators of psychosocial interventions on quality of life, and emotional and social function in patients with cancer: an individual patient data meta-analysis of 22 RCTs. Psychooncology..

[41] Dittus K, Toth M, Priest J, O’Brien P, Kokinda N, Ades P (2020). Effects of an exercise-based oncology rehabilitation program and age on strength and physical function in cancer survivors. Support Care Cancer.

[42] Leclerc AF, Slomian J, Jerusalem G, Coucke P, Bury T, Deflandre D, Devos M, Bruyère O, Foidart-Dessalle M, Kaux JF, Crielaard JM, Maquet D (2018). Exercise and education program after breast cancer: benefits on quality of life and symptoms at 3, 6, 12, and 24 months' follow-up. Clin Breast Cancer.

[43] Rammant E, Decaestecker K, Bultijnck R, Sundahl N, Ost P, Pauwels NS, Deforche B, Pieters R, Fonteyne V (2018). A systematic review of exercise and psychosocial rehabilitation interventions to improve health-related outcomes in patients with bladder cancer undergoing radical cystectomy. Clin Rehabil.

[44] Rath HM, Ullrich A, Otto U, Kerschgens C, Raida M, Hagen-Aukamp C, Koch U, Bergelt C (2016). Psychosocial and physical outcomes of in- and outpatient rehabilitation in prostate cancer patients treated with radical prostatectomy. Support Care Cancer.

[45] Silver JK, Baima J, Newman R, Galantino ML, Shockney LD (2013). Cancer rehabilitation may improve function in survivors and decrease the economic burden of cancer to individuals and society. Work.

